# The Importance of Human FcγRI in Mediating Protection to Malaria

**DOI:** 10.1371/journal.ppat.0030072

**Published:** 2007-05-18

**Authors:** Richard S McIntosh, Jianguo Shi, Richard M Jennings, Jonathan C Chappel, Tania F de Koning-Ward, Tim Smith, Judith Green, Marjolein van Egmond, Jeanette H. W Leusen, Maria Lazarou, Jan van de Winkel, Tarran S Jones, Brendan S Crabb, Anthony A Holder, Richard J Pleass

**Affiliations:** 1 Institute of Genetics, Queen's Medical Centre, University of Nottingham, United Kingdom; 2 Division of Parasitology, National Institute for Medical Research, Medical Research Council, London, United Kingdom; 3 Medical Research Council Technology, London, United Kingdom; 4 Walter and Eliza Hall Institute of Medical Research, Parkville, Victoria, Australia; 5 Department of Molecular Cell Biology and Immunology, VU Medical Centre, Amsterdam, Netherlands; 6 Immunotherapy Laboratory, Department of Immunology, University Medical Centre Utrecht, Utrecht, Netherlands; 7 Genmab, Utrecht, Netherlands; Center for Global Health and Diseases, United States of America

## Abstract

The success of passive immunization suggests that antibody-based therapies will be effective at controlling malaria. We describe the development of fully human antibodies specific for Plasmodium falciparum by antibody repertoire cloning from phage display libraries generated from immune Gambian adults. Although these novel reagents bind with strong affinity to malaria parasites, it remains unclear if in vitro assays are predictive of functional immunity in humans, due to the lack of suitable animal models permissive for *P. falciparum.* A potentially useful solution described herein allows the antimalarial efficacy of human antibodies to be determined using rodent malaria parasites transgenic for P. falciparum antigens in mice also transgenic for human Fc-receptors. These human IgG1s cured animals of an otherwise lethal malaria infection, and protection was crucially dependent on human FcγRI. This important finding documents the capacity of FcγRI to mediate potent antimalaria immunity and supports the development of FcγRI-directed therapy for human malaria.

## Author Summary

Malaria rivals HIV and tuberculosis as the world's most deadly infection killing a child every 30 seconds. Antibodies and their receptors (Fc-receptors) have been shown to be vital for the development of protective immunity, and as such they act as correlates of protection in studies aimed at defining the best antigens to incorporate into current vaccines. Understanding antibody types and Fc-receptors that optimally induce immunity is therefore vital to developing the best vaccines. Surrogate markers of antibody efficacy currently rely on in vitro assays that are laborious and difficult to reproduce. It remains unclear if such in vitro assays are predictive of functional immunity in humans due to the lack of suitable animal models permissive for *Plasmodium falciparum.* Here, we create a transgenic in vivo mouse model that has significant advantage over the use of new world primates, the only other model for human malaria. We demonstrate that this model defines an Fc-dependent mechanism of parasite destruction that cannot be assessed in current in vitro assays. The model provides both a test for therapeutic antibody efficacy prior to clinical trials in humans and an important tool in malaria vaccine development.

## Introduction

Malaria continues to kill approximately 2–3 million people each year [1]. The success of passive immunization in humans and animals suggests that immunoglobulin (Ig)-based therapies could be effective [2,3]. Manipulating antibody (Ab) genes allows the design of Ig with defined class and specificity, targeting protective epitopes on the parasite surface [4,5]. An appropriate target is the 19-kDa C-terminal region of merozoite surface protein 1 (MSP1_19_). This polypeptide displays limited sequence polymorphism possibly because the structure is constrained by its function [6], is expressed by all vertebrate asexual life-cycle stages [7], and acts as a major target of the erythrocyte invasion-inhibitory Ab response in individuals immune to Plasmodium falciparum malaria [8]. Novel genetic approaches, including linkage-group selection, have also identified MSP1 as an important target of immunity [9].

The mechanisms whereby Ig mediates protective immunity in malaria are less clear. The importance of Fc-receptor (FcR) subunits in malaria immunity has been studied in animals with FcR deletions. Although informative, these gene-deficient mouse models may not always mimic the human immune condition, due to differences in FcR biology and an apparent lack of true homologues [5]. Studies examining the role of FcR in immunity to parasites have made use of FcRγ-chain knockout mice [10,11]. The γ-chain, a subunit common to FcγRI, FcγRIIIa, FcɛRI, and FcαRI, is required for efficient cell surface expression and signal transduction. Consequently, FcRγ^−/−^ mice are unable to elicit phagocytosis or Ab-dependent cell-mediated cytotoxicity reactions through these receptors. Two recent studies with rodent malarias in the FcRγ^−/−^ have proved controversial, with one study showing a crucial role for FcR-mediated Ab-dependent phagocytosis in host resistance to blood-stage Plasmodium berghei XAT infection [10], and another study with Plasmodium yoelii concluding that the protective effects of Ab probably arise through FcR-independent mechanisms [11]. However, these studies ignore two important possibilities. Firstly, there might be other, as yet unidentified, FcR involved in the observed response, and secondly, the α-chain of many FcR may associate with other signaling proteins other than the common γ-chain. With this in mind, it is interesting to note that mouse IgG3-opsonized Cryptococcus neoformans can still be phagocytosed by macrophages from FcRγ^−/−^ mice [12]. This effect is probably mediated via an undefined FcR without requiring γ-chain for function because, of the known FcR, only murine FcγRI binds mouse IgG3, as demonstrated by transfection studies [13]. Secondly, FcRγ-chain-deficient mice were found to express partially functional FcγRI in more recent mouse knockouts [14,15]. It is now known that the γ-chain of FcγRI can mediate MHC class II Ag presentation without active γ-chain signaling [16], and that the α-chain can interact with Periplakin to control receptor endocytosis and IgG binding capacity [17]. These potential drawbacks to the rodent FcγRI knockout model led us to investigate the possibility of using human FcR transgenic mice to investigate Ab function with relation to malaria.

Antibodies have been shown to be vital for the development of protective immunity, and as such they act as correlates of protection in studies aimed at defining the best antigens to incorporate into current vaccines. Understanding which Ab and FcR combination optimally induces immunity is therefore vital to developing the best vaccines. Surrogate markers of Ab efficacy currently rely on in vitro assays that are laborious and difficult to reproduce and it remains unclear if such in vitro assays are predictive of functional immunity in humans due to the lack of suitable animal models permissive for P. falciparum [18,19]. By using rodent malarias transgenic for P. falciparum antigens in mice also transgenic for human FcRs, we have created a novel model that more fully mimics P. falciparum infection in humans, while providing an alternative to nonhuman primates for assessing the efficacy of anti–P. falciparum Abs prior to clinical trials in humans.

Here, we create novel fully human Abs specific for P. falciparum MSP1_19_ by Ab repertoire cloning from phage display libraries generated from malaria-immune Gambian adults. Using this unique dual transgenic approach, we were able to show that these human Abs are completely protective in vivo, and that this effect was crucially dependent on human FcγRI (CD64).

## Results

### Development of Antibody Repertoire Phage Display Libraries from Malaria-Exposed Donors

**Figure 1:**
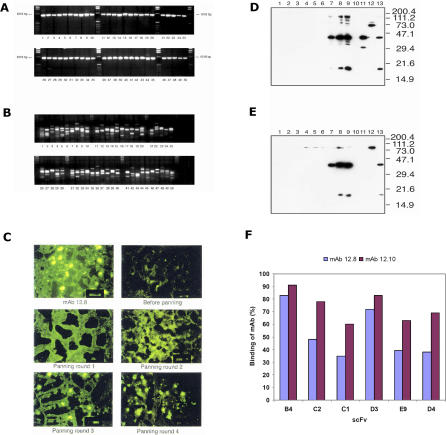
Characterization of scFvs Derived from Phage Libraries Estimation of diversity of library scFv inserts by BstNI digestion. Figure shows restriction digest patterns of 50 randomly selected scFvs from the October library. (A) 50 inserts of the V_H_–V_K_ scFvs amplified by PCR screen from 50 randomly selected colonies. (B) The same V_H_–V_K_ scFv inserts after digestion with BstNI. A large number of different digestion patterns are seen, suggesting a high degree of diversity among the library. (C–E) Binding of polyclonal scFv from panning rounds 1–4 to P. falciparum by IFA [20]. IFA was carried out using acetone-fixed parasites, and the polyclonal scFv tested against the T9–96 and FCB-1 strains of P. falciparum at 20 μg/ml [20]. MAb 12.8 was used as a positive control and polyclonal scFv prepared from the unpanned libraries used as negative controls. Immunoblots of polyclonal scFv from fourth round of panning binding to P. falciparum strain T9/96 (D) or FCB-1 (E) merozoites [20]. Lane 1, scFv March library unpanned; lane 2, scFv October library unpanned; lane 3–6, scFv from fourth round of panning with unprocessed merozoites; lane 7–8, scFv from fourth round of panning with processed merozoites; lane 9, scFv from fourth round of panning with recombinant MSP1_19_-GST; lane 10, scFv D1.3 (anti-hen's egg lysozyme); lane 11, scFv X509 (anti-MSP1_33_); lane 12, scFv 89.1 (anti-MSP1_83_); lane 13, mAb 12.8 (anti-MSP1_42_ and MSP1_19_). (F) Inhibition of binding of mAbs 12.8 and 12.10 to MSP1_19_ by scFvs derived from the fourth round of panning by competition ELISA [20].

We describe the construction of two phage display libraries derived from blood donations from malaria-exposed donors. The combined size of the two libraries (~1.3 × 10^9^ members) was sufficiently large to anticipate high affinity antibody fragments (unpublished data) [20]. PCR and BstNI fingerprinting of 100 randomly selected clones and their sequencing confirmed that they were derived from a wide range of different variable gene families (Figure 1A–1B). In additional experiments to estimate diversity, a number of clones were randomly sequenced from the unpanned libraries and found to be very diverse and derived from a wide range of different V gene families (unpublished data) [20]. Four rounds of panning with recombinant MSP1_19_-GST yielded scFvs capable of strong binding to P. falciparum parasites by immunofluorescence (IFA) (Figure 1C). Immunoblotting confirmed that polyclonal scFv from later rounds of panning did bind native MSP1 from T9/96 and FCB-1-derived P. falciparum merozoites (Figure 1D–1E). Intriguingly, panning against whole merozoites in which MSP1 processing had been allowed to proceed, gave almost identical patterns recognizing MSP1_19_ and MSP1_42_ to that seen when libraries were panned on recombinant proteins. Additionally, the close sequence similarity between scFvs obtained by panning with either processed merozoites or recombinant MSP1_19_ suggests that natural antibody responses to the merozoite after secondary processing are directed more toward MSP1_19_ than any other antigen remaining on the merozoite surface.

**Figure 2:**
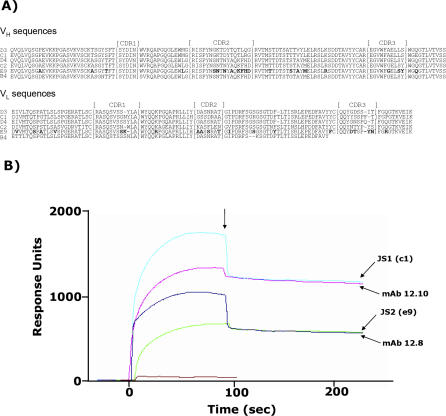
Sequencing of V Genes and SPR Analysis (A) Amino acid sequences of MSP1_19_-binding scFvs. Sequences of six selected scFvs obtained by panning phage display libraries with recombinant MSP1_19_ (C1) or P. falciparum merozoites in which secondary processing had been allowed to proceed (E9). Amino acids in bold represent residues in the E9 sequence differing to C1. (B) SPR association and dissociation curves of Ab binding to MSP1_19_ immobilized on a CM5 sensor chip. Abs were injected into flow at time 0 and replaced with buffer at the point indicated by vertical arrow [24].

Twenty individual MSP1_19_-binding scFv clones selected on the basis of their binding to MSP1_19_-GST by ELISA, their variety of restriction fragment patterns on BstNI digestion, and their ability to inhibit the binding of anti–P. falciparum MSP1_19_ monoclonal antibodies (mAbs) 12.8 and 12.10 in competition ELISAs (Figure 1F) were sequenced. mAb 12.8 and 12.10 have been shown to inhibit erythrocyte invasion in vitro by P. falciparum merozoites [21,22]. Of these 20 clones, six different scFv sequences were found (Figure 2A). None of the six scFvs inhibited erythrocyte invasion in vitro [20]. We therefore engineered the most promising scFvs into fully human antibodies since the presence of the Fc may potentiate inhibition of erythrocyte invasion, a likely prediction given that Fab and F(ab′)_2_ fragments of mAb 12.10 do not retain invasion-inhibitory properties [23]. Intriguingly, we have recently isolated scFv to other key malaria antigens from this library, including EBA-175 and EMP1 (unpublished data).

### Development of Two Fully Human IgG1s Recognizing P. falciparum MSP1_19_

**Figure 3:**
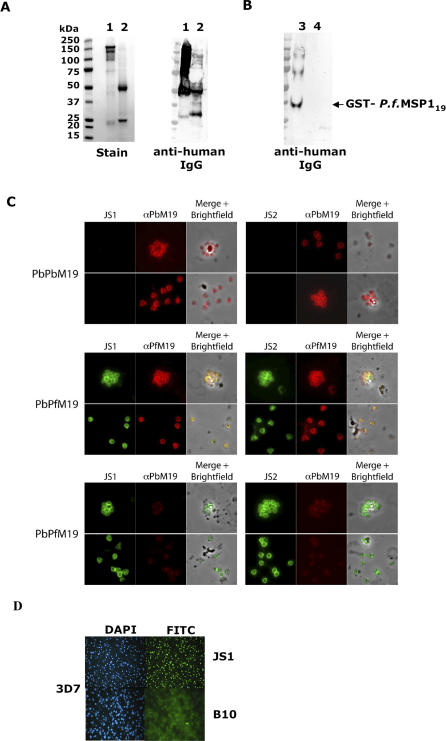
Characterization of Purified Abs (A) 5 μg purified anti-MSP1_19_ human IgG1 (JS1) was subjected to SDS-PAGE under nonreducing (lane 1) or reducing (lane 2) conditions on 4%–15% polyacrylamide gradient gels and stained with Simply Blue or immunoblotted with anti-human IgG-HRP. (B) Under nonreducing conditions and after transfer to nitrocellulose, the human anti-MSP1_19_ IgG1 (JS1) detects the recombinant GST-MSP1_19_ fusion protein (lane 3) but not the GST alone control (lane 4). Localization of MSP1_19_ by IFA. (C) Schizont- and merozoite-stage parasites from the transgenic PbPbM19 and PbPfM19 lines were incubated with human Abs JS1 or JS2 (1:100), rabbit αPbM19 (1:1,000), or αPfM19 (1:1,000). After incubation with goat anti-rabbit Alexa-conjugated Ig (1:1,000) and FITC-conjugated anti-human IgG Fc (1:200), slides were washed and mounted in Vectrashield anti-fade. Parasites were visualized by fluorescence microscopy ×100 magnification, with the same fields photographed using filters to detect Alexa and FITC. (D) JS1 reactive with MSP1_19_ on methanol-acetone-fixed smears of merozoites and erythrocytes infected with P. falciparum (strain 3D7) ×40 magnification. JS2 gave similar results. No specific fluorescence was detected with an irrelevant human IgG1 (B10) recognizing MSP1_19_ from P. yoelii [24].

We subcloned the variable genes derived from two promising scFvs c1 and e9 and linked them to human IgG1 constant domains in expression vectors as previously used to successfully generate chimeric human anti–P. yoelii MSP1_19_ IgG1s [24]. Both antibodies, now termed JS1 (derived from phage c1) or JS2 (derived from phage e9), when purified from CHO-K1 transfectant culture supernatants contained polypeptides of the expected sizes on SDS-PAGE and the anticipated reactivities with isotype-specific antibodies (Figure 3A). JS1 and JS2 recognized a P. falciparum MSP1_19_-GST fusion protein (Figure 3B), and by indirect IFA, both antibodies produced a characteristic pattern of MSP1 reactivity in schizonts, merozoites, and ring-stage parasites from P. berghei parasites transgenic for P. falciparum MSP1_19_ (PbPfM19), but not to the control transgenic line PbPbM19 previously engineered to integrate in an identical manner the homologous P. berghei MSP1_19_ sequence (Figure 3C) [25]. The use of P. berghei and P. falciparum MSP1_19_-specific antibodies (αPbM19 and αPfM19) allowed confirmation of the genotype of the transgenic lines while demonstrating that both JS1 and JS2 colocalize only with αPfM19 detecting reagent (Figure 3C, merge + brightfield). This novel rodent malaria model thus provides an alternative to nonhuman primates for assessing and monitoring the efficacy of P. falciparum MSP1_19_-based human antibodies in mice transgenic for human Fc-receptors [25]. In addition, both JS1 and JS2 bound to the 3D7 P. falciparum reference strain (Figure 3D).

Importantly, surface plasmon resonance (SPR) analysis revealed no reduction in affinity for MSP1_19_ for either JS1 or JS2 when compared with mouse mAbs 12.8 and 12.10 (Figure 2B). However, JS2 did have a reduced on-rate and therefore a lower overall affinity for P. falciparum MSP1_19_ when compared with JS1, a finding that may explain the reduced potency seen with JS2 in vivo. Despite comparable affinities for MSP1_19_, neither JS1 nor JS2 inhibited MSP1 processing when compared to mAb 12.8 (unpublished data, available on request).

### JS1 and JS2 Are Fully Functional Human IgG1 Antibodies

**Figure 4:**
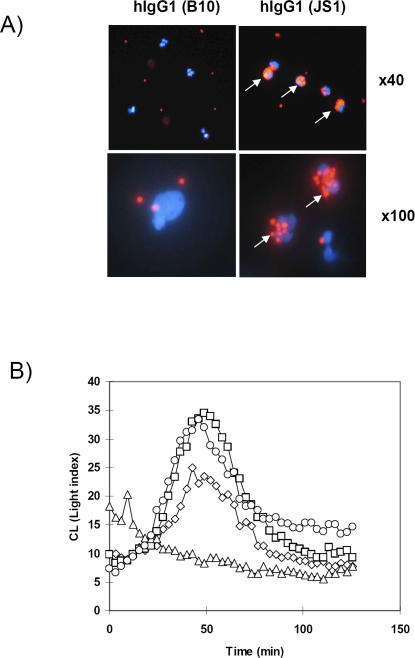
JS1 and JS2 Are Fully Functional Human Antibodies (A) Human neutrophil-mediated phagocytosis of GST-*Pf*MSP1_19_ coated fluorescent 1-μm microspheres by JS1. A control human IgG1 recognizing the homologous GST-MSP1_19_ from P. yoelii was unable to opsonize beads and no ingestion was observed. Phagocytosed beads (red) were visualized in the cytoplasm of neutrophils (arrowed) whose nuclear DNA was counterstained in blue by DAPI. (B) Stimulation of neutrophil NADPH oxidative bursts using JS1 and JS2 attached to GST-MSP1_19_-coated microtiter plates. Chemiluminescence (CL; arbitary units) was induced by anti-*Pf*MSP1_19_ human IgG1 JS1 (⋄), JS2 (○), human IgA1 (□), or no antibody (▵). All antibodies at 1 × 10^−7^ M. Data are presented as mean arbitary units from duplicate wells with neutrophils from a single donor.

We assessed the ability of these novel reagents to phagocytose MSP1_19_-coated fluorescent microspheres (Figure 4A). Beads opsonized with JS1 Abs specific for MSP1_19_ from P. falciparum could be detected within human neutrophils. The capacity to ingest these “pseudomerozoites” was enormous with up to 20 beads being detected in some neutrophils. We also assessed the ability of these novel reagents to elicit an oxidative burst and degranulation in human neutrophils (Figure 4B). When attached to GST-*Pf*MSP1_19_-coated plates at equimolar concentrations, an epitope matched human IgA1 recently constructed from the same variable genes as in JS1 (unpublished data) was consistently the most efficient at inducing respiratory bursts, although both JS1 and JS2 were also very effective. Since uninduced human neutrophils do not express many molecules of FcγRI, phagocytosis and respiratory bursts in this instance most likely occur through FcγRIIA and/or FcγRIIIB.

### Epitope Mapping of JS1 and JS2 Binding Sites on MSP1_19_

**Figure 5:**
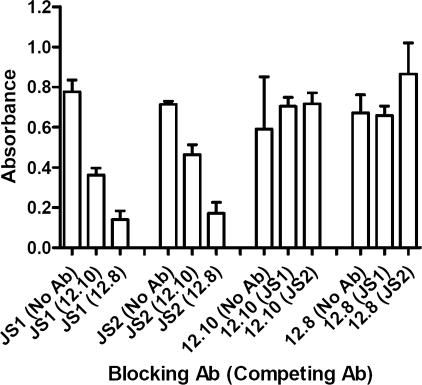
Inhibition of Binding of mAbs 12.10 and 12.8 by Fully Human Anti-MSP1_19_ IgG1s (JS1 and JS2) by Competition ELISA The binding of 12.8 is reduced by over 60% and the binding by 12.10 reduced by 30% suggesting that JS1 and JS2 compete with mAbs 12.8 and 12.10 for similar or overlapping epitopes. mAb 12.8, JS1, and JS2 were used at 0.5 μg/ml and mAb 12.10 at 0.05 μg/ml. Similar results were also observed when saturating concentrations of antibodies were used.

**Figure 6:**
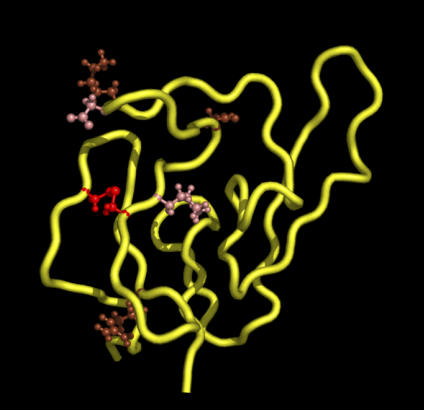
Epitope Mapping of JS1 and JS2 Binding Sites Shows the location in the three-dimensional model of P. falciparum MSP1_19_ of residues in the first epidermal growth factor domain, which on mutation affect binding by JS1 or JS2. Mutation of Cys^28^ shown in red completely ablated binding of both mAbs (12.10 and 12.8) and JS1 or JS2. Mutation of the partnering Cys^12^, also shown in red, while ablating binding by the murine mAbs, had no effect on the binding by JS1 or JS2. Arg^20^ and Asn^33^ in salmon had intermediate effects on binding as determined by SPR analysis when mutated to more neutral or negatively charged side-chains (see Table 1). Three further substitutions at Lys^40^, Lys^29^, and Asn^39^ seen in brown had minor effects on binding when the interaction was studied by ELISA. The model of P. falciparum MSP1_19_ was generated by PyMol using atomic coordinates available from NCBI under accession number PDB: 1CEJ.

A number of approaches were used in order to identify the amino acid residues in MSP1_19_ contributing to the binding of JS1 and JS2. A competition ELISA was designed to determine whether JS1 or JS2 could compete with mAbs 12.8 or 12.10 for binding sites on MSP1_19_ (Figure 5). Binding of either JS1 or JS2 reduced the binding to MSP1_19_ of mAbs 12.8 and 12.10 by ~60% and 30%, respectively, suggesting that JS1 and JS2 compete with mAbs 12.8 and 12.10 for similar epitopes. JS1 and JS2 may therefore be described as blocking antibodies since they inhibit the binding of invasion inhibitory mAbs. Intriguingly, the converse experiment with 12.10 and 12.8 failed to inhibit binding of JS1 or JS2, suggesting that the fully human antibodies may bind MSP1_19_ with a higher affinity and/or that the epitopes seen are nonidentical but overlapping. In order to identify the amino acid residues involved in JS1 and JS2 binding, we made use of a panel of MSP1_19_ mutants available from a previous study investigating the binding of inhibitory and blocking mAbs [26]. Binding of Abs to the modified proteins was detected by ELISA and SPR analysis. Table 1 summarizes the effect of 11 single and six multiple amino acid substitutions on binding by JS1 and JS2 compared with the previously described effects on interaction with mAbs 12.10 and 12.8. Of these, only one amino acid substitution (Cys^28^→Trp) completely ablated binding by both JS1 and JS2. Cys^28^ forms a disulfide bond with Cys^12^, the mutation of which (Cys^12^→Ile) had no affect on binding by JS1 or JS2 but did completely prevent binding by the mAbs 12.10 and 12.8. Six further mutants had intermediate effects on binding by JS1 and JS2, with (Arg^20^→Glu) and (Asn^33^→Ile) being particularly prominent since no binding by SPR analysis was seen when these mutant proteins were passed over JS1 or JS2 at identical concentrations to wild-type protein (Figure 6 and Table 1). Three further substitutions at Lys^40^, Lys^29^, and Asn^39^ only had minor affects on binding. No differences in gross binding (other than minor differences in affinity) could be seen to distinguish JS1 from JS2, despite significant variability (20 amino acid substitutions throughout each V_L_ and V_H_ gene) within the complementarity determining regions of their respective variable genes (Figure 2A and Table 1). These findings support our assertion that JS1 and JS2 bind to nonidentical but overlapping residues in the first epidermal growth factor domain of MSP1_19_, in common with mAbs 12.8 (Figure 6 and Table 1) and 12.10 (for which the epitope also has a contribution from the second epidermal growth factor domain).

**Table 1:**
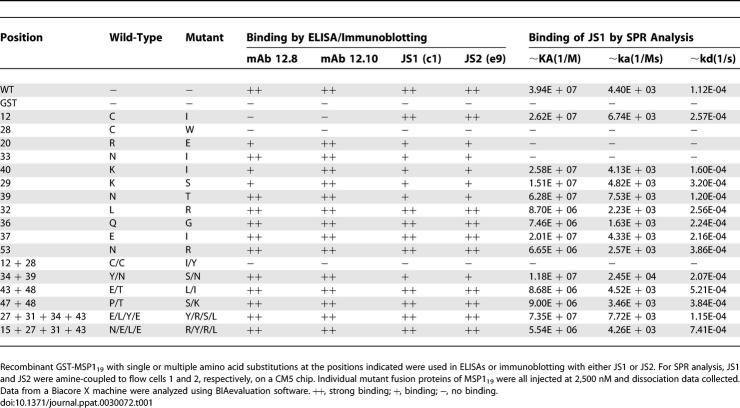
The Location of Amino Acid Residues and the Modifications Made within MSP1_19_ and Their Effect on the Binding by Fully Human IgG1 (JS1 and JS2) or Previously Characterized Mouse mAbs 12.8 and 12.10

### Suppression of Parasitemia by JS1 Is Critically Dependent on the Presence of Human FcγRI

**Figure 7:**
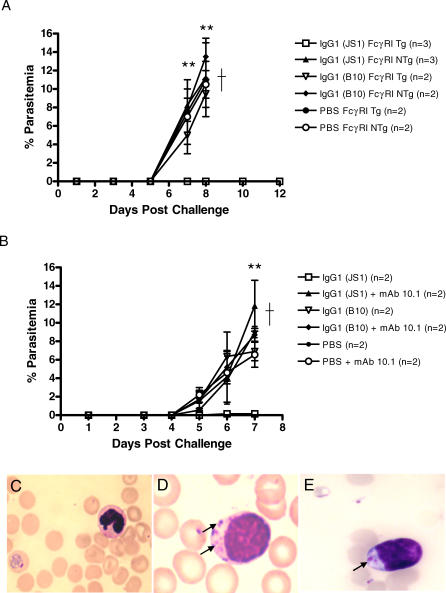
Course of a P. falciparum MSP1_19_ Transgenic P. berghei Infection in Mice (A) Groups of 2–3 FcγRI transgenic (Tg) or nontransgenic (NTg) littermates were injected i.p. with a total dose of 1.5 mg fully human anti–P. falciparum MSP1_19_ IgG1 (JS1), an irrelevant human IgG1 (B10) recognizing MSP1_19_ from P. yoelii or PBS. Similar results were obtained in two independent experiments. **Only groups of mice given JS1 in the FcγRI Tg were significantly different to all the other control groups with a *p* < 0.01. †, death of mice. (B) Repeat experiment in FcγRI Tr animals using a lower total dose (0.75 mg) of JS1. Coadministration of the blocking mAb 10.1 specific for the IgG1 binding site on human FcγRI abrogates the protection mediated by the passively administered JS1 antibody. Each point represents the geometric mean parasitemia of mice in each group at the time after i.p. challenge with 5,000 parasitized erythrocytes. Only those animals receiving the fully human anti–P. falciparum MSP1_19_ IgG1 Ab in a human FcγRI background survived an otherwise lethal infection; all the mice in the other groups with high parasitemias were killed on either day 7 or 8. Similar results were obtained in two independent experiments. **Only groups of mice given JS1 in the FcγRI Tg were significantly different to all the other control groups with a *p <* 0.01. †, death of mice. (C–E) ×100 magnification of Giemsa-stained smears of blood taken from control animals (C) and FcγRI Tg animals treated with JS1 (D and E). Note the presence of phagocytosed merozoites within the cytoplasm of cells displaying mononuclear morphology (arrow).

In vivo experiments with P. berghei transgenic for P. falciparum MSP1_19_ demonstrated that three intraperitoneal (i.p.) inoculations of JS1 (total dose of 1.5 mg antibody) effectively suppressed a lethal blood stage challenge infection in mice transgenic for human FcγRI (Figure 7A). However, JS1 was nonprotective in nontransgenic littermates allowing us to conclude that FcγRI recruitment by this fully human anti-MSP1_19_ IgG1 is crucial for therapeutic effectiveness. A simple neutralization of MSP1_19_ function by some form of steric hindrance is therefore insufficient to bring about protection by this human IgG1. As expected, human IgG1 (B10) recognizing MSP1_19_ from P. yoelii was completely ineffective at controlling parasitemias, confirming that binding of transgenic FcγRI by human IgG1 is insufficient to induce protection in the absence of specific high-affinity antigen binding (Figure 7A).

Further experiments with JS1 could show that a total dose of as little as 0.75 mg of antibody could result in effective parasite clearance (Figure 7B). Importantly, protection could be reversed with passive administration of mAb 10.1 (Figure 7B), a mouse mAb that binds to human FcγRI blocking the human IgG1 binding site, and allowing us to conclude that immune protection is crucially dependent on the presence of the human FcγRI transgene and not endogenous murine FcγRs. JS2 was equally effective at controlling malaria at the higher Ab dose of 1.5 mg/animal but was unable to prevent the development of parasitemia at lower doses (≤0.75 mg/animal) suggesting that subtle differences in affinity or fine-specificity can ultimately impact significantly on in vivo efficacy (unpublished data). Such a prediction could not have been envisaged from in vitro assays while confirming the importance of our in vivo model for screening antibodies that otherwise appear promising from initial in vitro screens.

Although we were unable to detect free merozoites or infected erythrocytes from FcγRI transgenic animals treated with JS1, we did notice that many circulating phagocytes with classical monocyte type morphology contained Giemsa-staining merozoites within their cytoplasm (Figure 7C versus 7D and 7E).

There was no observable difference in the ability of the FcγRI transgenic mice versus nontransgenic animals in rates of clearance of human antibodies, work that has also recently been confirmed in animals lacking all FcγRs, suggesting that the neonatal FcR is wholly responsible for the kinetics of clearance (unpublished data).

## Discussion

We describe the development of the first fully human IgG1 with specificity for an epitope on MSP1_19_ from *P. falciparum,* for dissecting human FcR mechanisms involved in immunity to human malaria. These antibodies may also be employed in the clinic as therapeutically useful entities. These novel reagents were generated through cloning of human Ig variable domains from human combinatorial phage display libraries derived from malaria immune Gambians and their engraftment onto human constant region genes, for expression in mammalian cells. Although fully human antibodies have been generated to other P. falciparum antigens, the Fc-receptors through which these antibodies confer their protective effects have not been elucidated [27].

The engineered Abs recognized parasites in infected erythrocytes and triggered potent human FcγRI-mediated protection in a novel Ab testing system involving the use of P. berghei parasites transgenic for P. falciparum MSP1_19_ and mice transgenic for human FcRs. The human Abs bound to nonidentical but overlapping residues in the first epidermal growth factor domain of P. falciparum MSP1_19_ and with similar affinities to mouse mAbs, 12.10 and 12.8, already known to inhibit erythrocyte invasion in vitro. Importantly, the inability of human IgG1 to protect in nontransgenic mice indicates that mere blocking of MSP1_19_ function by some form of steric hindrance or interference with MSP1_19_ processing is insufficient to bring about protection, and that the presence of the Fc region is crucial, since this allows the recruitment of FcγRI-mediated parasite clearance. In contrast to mAb 12.10 and 12.8, neither JS1 nor JS2 could inhibit MSP1 processing in vitro (unpublished data), suggesting that their efficacy in vivo is wholly dependent on Fc-mediated pathways. It will be informative to determine if subcloning the V genes onto alternative Fc scaffolds, such as IgG3 and IgA, which having larger Fc regions and greater flexibility to the IgG1 created here, would confer processing inhibitory activity. Interestingly, the JS1 and JS2 antibodies would be classified as blocking antibodies that favor erythrocyte invasion using an in vitro assay, but here the in vivo assay shows that they are important in parasite destruction.

Since intact 12.10 and 12.8 can inhibit MSP1 processing and invasion of erythrocytes in in vitro assays and Fabs and F(ab′)_2_ cannot, it is likely that the Fc portion of the antibody contributes in some way to this process. The Fc is of significant size (50 kDa) and is attached to the rest of the antibody via a flexible hinge that allows much wagging and flexibility in this region. Indeed the flexibility of the Fc is known to make an important contribution to function; therefore, it is not inconceivable that it may interfere with invasion by some form of steric hindrance of the merozoite. An alternative explanation not requiring FcR-bearing effector cells, is that complement plays a role in this process since only an intact antibody can activate the complement cascade and this occurs through the Fc-region, while explaining the lack of effect with Fabs or F(ab′_2_). However, inhibition of invasion assays are done with heat-inactivated serum or in the presence of Albumax in the place of serum, suggesting that complement does not play a role, at least in vitro.

These unique Abs allowed us to show a specific mechanism of action in vivo, in experiments using mice transgenic for human FcγRI. Such in vivo experiments are not possible in humans with P. falciparum malaria. Hence, recombinant human Abs engineered as described will be useful in correlating particular epitopes on MSP1_19_ with protective immunity, as an aid to vaccine design, and will form the bases of effective in vivo assays prior to clinical trials in humans. Until now, work on human malaria parasites has made use of polyclonal Abs purified from immune sera in neutralization tests in vitro [18,19]. These antibody-dependent cellular inhibition experiments are technically demanding and far from optimal since sera contain a mixture of Abs, some with inappropriate specificities (such as blocking Abs) and the potential to trigger inhibitory FcR through intracellular immunoreceptor tyrosine inhibition motif signaling. The recombinant antibodies described herein are of a defined class and specificity and offer reproducible standards for such assays in an in vivo system, a model that has hitherto not been possible. The P. berghei mouse transgenic combination is a good model for P. falciparum in humans since mouse IgG2a binds with similar affinity to human monocytes as human IgG1, indicating that the transgenic receptor would be fully occupied with mouse IgG2a in vivo and accurately reflecting the situation in humans with IgG1 [28]. We believe that because of the obvious limitations to primate and SCID mouse studies, the transgenic approach described here is the best current model in which to address human antibody efficacy and function.

Although Abs (in particular of the IgG1 and IgG3 classes) from clinically immune individuals are without question involved in immunity to malaria, the role of their cognate FcRs has been investigated less thoroughly. FcγRI expression in these transgenic mice is limited to cells of the myeloid lineage, including monocytes and dendritic cells and can be upregulated by several cytokines including IL-10 and IFN-γ, both of which are implicated in immunity to malaria [29,30]. Previous work has highlighted monocytes in Ab-dependent killing of P. falciparum asexual blood stages and implicated the involvement of FcγRII and FcγRIII [18]. From a therapeutic bioavailability standpoint triggering FcγRII may not be ideal. This is because both IgG1 and IgG3 can bind FcγRs on cells that do not directly kill parasites, including platelets, B cells, endothelial cells, and even placental tissue, resulting in the triggering of inhibitory FcγRII receptors [4,5]. This might benefit the parasite, analogous to the effect on tumor cell growth, in which passively administered anti-tumor Abs enhanced tumor cell growth through FcγRIIb [31]. That this is indeed the case for malaria has recently been shown in an elegant study in FcγRIIb-deficient mice, which have increased clearance of Plasmodium chabaudi malaria and develop less severe disease [32]. The same study went on to show that polymorphic variants of human FcγRIIb resulting in loss-of-function are common in African individuals who also show enhanced phagocytosis of parasites. Unfortunately, this study used pooled immune serum and whole parasites, and the contribution of individual IgG subclasses and the antigens driving FcγRIIb signaling could not be investigated. The epitope-specific reagents described in this study would allow these questions to be answered for fully human antibodies. For example, we are in the process of mutating our anti-MSP1_19_ human IgG1s to make more potent antimalarial antibodies by changing residues in the Fc that optimize binding to the activatory receptors, including FcγRI, at the expense of binding to the FcγRIIb inhibitory receptor [33].

Merozoite killing can also be mediated by neutrophils [present study and 24,34]. Neutrophils are the most populous leucocyte in blood and can express FcγRI on activation with IFN-γ or G-CSF and targeting Candida albicans toward neutrophil FcγRI results in potent fungicidal activity in vivo [35,36]. However, we do not observe any increase in expression of the FcγRI transgene on neutrophils from animals infected with malaria parasites and therefore conclude that protective immunity manifested by this IgG1 is mediated through monocytes (unpublished data and Figure 7).

It is known that the α-chain of FcγRI can mediate protective signaling events either through the common α-chain or via Periplakin [17]. The availability of human FcγRI-transgenic animals deficient in Periplakin will permit the delineation of which signaling pathway is important in protection to malaria. FcγRI represents the only FcγR with a well-documented capacity to facilitate immunological memory in vivo [37,38]. This may be crucial in the context of malaria where the inability to induce or maintain long-term memory responses is likely to pose major problems for the development of effective vaccines [39].

Although no crystal structure exists for the interaction of human FcγRI with IgG1, the location has been inferred by comparison with known structures for the association of IgG1 with FcγRIII [40]. Such analyses consistently highlight an extended hydrophobic area formed at the interface between the two N-terminal domains with contacting residues within the D1/D2 connector, the B/C, C/E, and F/G loops of the EC2 domain. This region shows great variability between the membrane-anchored version of FcγRI, encoded by FcγRIa, and the FcγRIb and FcγRIc genes which are predicted to encode secreted forms that suggest differences in ability to bind IgG1. The role of these variant genes in malaria is unknown. Although polymorphisms in FcγRIIa, FcγRIIIb, and more recently FcγRIIb, have been implicated in susceptibility to severe malaria, no associations with FcγRIa, or its associating subunits, have yet been made [32,41–43]. It will be important to determine if our fully human IgG1s are also protective in mice transgenic for other human FcγRs, including FcγRIIa.

By searching single nucleotide polymorphism (http://www.ncbi.nlm.nih.gov/SNP) and trace databases, we identified FcγRIa-specific polymorphic residues both in this region of EC2 and in the cytoplasmic signaling domain that might affect the outcome of malaria in human populations. By creating mice transgenic for these variants, our humanized model for P. falciparum malaria provides an opportunity for testing experimentally and in vivo the effect of these polymorphisms on the efficacy of antibody function in relation to malaria. The ability to predict how antibodies will work in groups of patients with particular FcγR polymorphisms would allow targeted and tailored therapy for these expensive reagents.

A clearer understanding of the role for individual FcγRs in malaria immunity is vital if Abs are to be used successfully as therapeutic entities. Although human anti-MSP1_19_ IgG1 is clearly effective in passive immunization, its usefulness remains to be tested in rodents or humans with already well-established parasitemias. That they have the potential to be extremely useful in such settings is supported by proof-of-principle studies showing that passive transfer of polyclonal IgG can reduce existing parasitemias in children and adults [2,44].

A potential drawback to using IgG1 for malaria therapy in humans is the high level of nonspecific IgG1 in the hypogammaglobulinemia induced shortly after infection [4,5,45]. Such Abs might compete with passively administered IgG1 for FcγRI occupancy, and therefore phagocyte recruitment. The presence of pre-existing IgG may explain why large doses of Ab have been required to neutralize parasites both in vitro and in vivo [46]. Extrapolating to humans, the effective dose of 0.75 mg/animal seen with JS1 compares well with the required dosage of Palivizumab (15–75 mg/kg) known to protect at-risk infants from infection with respiratory syncytial virus [47]. A potential solution would be to engineer increased affinity for FcγRI into our anti-MSP1_19_ IgG1, for example, by introducing more hydrophobic residues in place of Leu^235^, Pro^329^, and Leu^328^ that would bind to FcγRI in preference to pre-existing IgGs, thus facilitating the use of lower dosages [40].

Given increasing problems with resistance to antimalarial drugs, a vaccine against malaria has become the ultimate goal. Unfortunately, its development has been beset with problems, and alternative strategies to treat malaria are urgently sought. Here we have shown that the passive delivery of recombinant Abs should be considered important adjuncts to more traditional vaccine approaches, since Abs have the potential to act both as therapies and as vehicles for the optimal delivery of antigens in vaccination.

## Materials and Methods

### Phage libraries and selection procedures.

Two phage-display combinatorial immune antibody libraries were created from peripheral blood mononuclear leucocytes derived from 20 Gambian donors during the dry season (March 1997 library, low prevalence of clinical malaria) or at the end of the wet season (October 1997 library, high prevalence of clinical malaria). For this study, informed consent was obtained with guidelines of the Gambian Ministry of Health and the Medical Research Council, whose ethical review committees approved all protocols. From these, ten donors were selected at random on the basis of previously noted high levels of anti-MSP1 IgG for library construction. The scFv libraries were constructed using standard sets of primers from pooled total RNA obtained from peripheral blood monocytes as described [20,48]. scFvs from the heavy- and light-chain repertoires were cloned sequentially into pHEN1H6 upstream of hexa-HIS and *c-myc* tags via SfiI/NotI restriction sites. The phage libraries were panned for binders using immunotubes (Nunc, Maxisorp, http://www.nuncbrand.com) and coated with recombinant MSP1_19_-GST [49]. Approximately 1 × 10^14^ phage from each of the two libraries were blocked in PBS containing 18% (w/v) skimmed milk powder and 100 μg/ml GST to prevent selection of anti-GST scFvs during panning, prior to addition to the coated immunotubes at 37 °C for 1 h. Bound phage were eluted into 100 mM triethylamine (pH 11.0), neutralized with 1 M Tris-HCl (pH 7.4), and then allowed to infect Escherichia coli TG1 host cells for amplification. After three further rounds of binding and amplification, 96 single clones were screened for binding to MSP1_19_ by: (a) direct ELISAs, (b) competition ELISAs with mAb 12.8 and 12.10, known to be potent inhibitors of erythrocyte invasion in vitro, (c) IFA to acetone-fixed P. falciparum–infected erythrocytes, and (d) immunoblotting, all as described previously [21–24].

### Construction of human antibodies.

The V_H_ genes derived from scFv clones c1 and e9 were amplified by PCR using the following pairs of forward (5′ACAGGCGCGCACTCCGAGGTGCAGCTG-3′) and reverse (5′ACCTGAGGAGACGGTGACCAGGGT-3′) primers and subcloned as BssHII/BstEII fragments into pVHExpress vectors, upstream of the γ1 constant region. V_L_ genes were amplified using sequence specific forward (c1, 5′-GGCGTGCACTCCGATATTGTGATGACCCAG-3′ and e9, 5′-GGCGTGCACTCCGATGTTGTGATGACTCAG-3′) and reverse primers (5′-GATCTCGAGACTCACGTTTGATCTCCA-3′) as an ApaLI/XhoI fragment into pVKExpress upstream of the human κ gene [24,50]. Chinese hamster ovary (CHO)-K1 cells were transfected with corresponding heavy- and light-chain plasmids and positive clones secreting MSP1_19_-specific IgG1 detected by ELISA or immunoblotting using goat anti-human IgG conjugated to horseradish peroxidase as described previously [24]. Two fully human antibodies were produced, JS1 and JS2, derived from scFvs c1 and e9, respectively. From large-scale cultures of both JS1 and JS2, human IgG1 was purified on HiTrap protein G-Sepharose (GE Healthcare, http://www.gehealthcare.com) by FPLC. The integrity and purity of the antibodies was verified on sodium dodecyl sulfate-polyacrylamide gel electrophoresis (SDS-PAGE) gels.

### Antigen binding and invasion–inhibitory assays.

For direct ELISAs, scFv supernatants from individual clones were added to wells of a microtiter plate (Nunc, Maxisorp) coated with MSP1_19_-GST (diluted to 1 μg/ml in coating buffer) after incubation in blocking solution (phosphate buffered saline/3% milk powder). The scFvs were allowed to bind for 1 h at 37 °C before three washes in PBS/0.1% Tween-20 and three further washes in PBS. 100 μl of 9E10 anti-*c-myc* hybridoma supernatant was added to each well. After 1 h the plate was washed as above and 100 μl of a 1/5,000 dilution peroxidase-conjugated goat anti-mouse antibody (Jackson ImmunoResearch Laboratory, http://www.jacksonimmuno.com) in blocking solution was added [20]. After 1 h the plate was washed as before and developed with Neogen K-BLUE substrate (http://www.neogen.com). For western blotting, T9/96 or FCB-1 P. falciparum merozoites were boiled in nonreducing sample buffer and extracts loaded into wells of a 15% SDS-PAGE gel prior to transfer to nitrocellulose membranes [20]. The membrane was incubated in blocking solution for 2 h before adding polyclonal scFv at 1 μg/ml for 1 h. The membrane was washed three times with PBS/0.1% Tween-20 and then incubated with 9E10 supernatant for 1 h on a rotating platform. A 1:25,000 dilution of peroxidase-conjugated goat anti-mouse antibody in blocking solution was added and incubated for 1 h followed by washes as above [20]. Membranes were developed using ECL chemiluminescent detection system (GE Healthcare). Selected Ni-NTA purified or polyclonal scFvs and intact antibodies were tested for their ability to bind acetone-fixed T9/96, FCB-1, and 3D7 P. falciparum or the P. berghei transgenic for P. falciparum MSP1_19_ (PbPfM19) by IFA as described previously [20,24,25]. Invasion and processing assays were carried out according to previously published protocols [21].

### Epitope mapping.

A number of approaches were used to map the antigenic structures recognized by the V genes cloned from scFvs c1 and e9. A competition ELISA was designed to determine if scFvs or intact Abs could compete with mAbs 12.8 and 12.10 for binding sites on the MSP1_19_ molecule. After coating in pre-blocked scFv or JS1/JS2 and washed as above, mAb 12.8 or 12.10 (0.5 μg and 0.05 μg/ml, respectively) diluted in blocking solution were allowed to bind. Bound mAb was then detected as described above [20]. A second approach, combining ELISAs, western blotting, and SPR analysis took advantage of an extensive panel of recombinant MSP1_19_ mutants derived by site-directed mutagenesis and used in an earlier study to map both inhibitory and blocking mAbs [26]. For a quantitative comparison of binding, human IgG1s (JS1 or JS2) were amine-coupled to each flow cell of a CM5 sensor chip. 2,500 nM of each MSP1_19_ mutant was then injected over each antibody and association and dissociation observed. Data from a BIAcore X machine were analyzed using BIAevaluation 3.0 software. Antibody binding to the panel of mutants was scored as follows: “++” indicates approximately the same amount of antibody binding to the mutant MSP1_19_ as to wild-type, “+” indicates reduced binding to the mutant compared to wild-type, and “−” indicates no detectable antibody binding to the mutant protein.

### Respiratory burst and phagocytosis assays.

A chemiluminescence-based neutrophil respiratory burst assay was conducted on plates coated with recombinant GST-*Pf*MSP1_19_ at 10 μg mL^−1^ as previously described [24]. 1 μm red fluorescent carboxylate-modified microspheres (Molecular Probes, http://probes.invitrogen.com) were covalently coupled with GST-*Pf*MSP1_19_ using 1-ethyl-3-(3-dimethylaminopropyl)-carbodiimide, hydrochloride (EDAC) as per manufacturer's instructions. 7.2 × 10^4^ beads were coated with 100 μg/ml of each antibody in PBS/10% FCS. After three washes, beads were resuspended in a final volume of 100 μl containing 5 × 10^4^ neutrophils purified as previously described [24]. Cells and beads were gently pelleted at 1,200 rpm and incubated for 30 min at 37 °C. Cells were then gently resuspended and smeared onto glass slides and allowed to dry prior to mounting in ProLong Gold antifade reagent with DAPI (Invitrogen, http://www.invitrogen.com). Phagocytosis was observed by immunofluorescence microscopy (Zeiss Axioskop 40, http://www.zeiss.com).

### Passive immunization and parasite challenge.

Because of the lack of an animal model for *P. falciparum,* human FcγRI transgenic mice have been developed that exhibit similar FcγRI cell distribution and expression patterns as in humans [29]. Tg Balb/c × Balb/c F1 mice 9- to 12-wk-old and bred under specific pathogen-free conditions were used. Nontransgenic littermates served as controls. Mice were screened for FcγRI expression by PCR of whole blood using forward (5′ AGATTTCACTGCTCCCACCA-3′) and reverse (5′ CACTTGCCCATCAACTGGA-3′) primers for human CD64 and by analysis of lysed whole blood on a FAC-Scan with FITC-conjugated anti-human FcγRI (unpublished data). Antibodies (0.25 or 0.5 mg/injection depending on experiment) or blocking mAb 10.1 (at 50 μg/injection, Serotec, http://www.ab-direct.com) were administered i.p. on day −1, day 0, and day +1 with respect to parasite challenge. Parasitized erythrocytes (5,000/mouse) derived from passaged mice infected with P. berghei parasites transgenic for P. falciparum MSP1_19_ were injected i.p. at least 3 h after antibody treatment on day 0 as previously described [24,25]. Parasitemia was assessed daily on Giemsa reagent-stained blood smears. Differences between groups were analyzed over two replicate experiments using the Mann Whitney test. A *p-*value < 0.01 was considered significant. All animal experiments were approved by the Home Office and performed in accordance with United Kingdom guidelines and regulations (PPL40/2753).

## Abbreviations

Ab: antibody
FcR: Fc receptor
IFA: immunofluorescence
Ig: immunoglobulin
i.p.: intraperitoneally
mAb: monoclonal Ab
MSP1: merozoite surface protein 1
PbPfM19: *Plasmodium berghei* parasites transgenic for *Plasmodium falciparum* MSP1_19_
SPR: surface plasmon resonance
